# Cardiac Tissue Damage in a Female Animal Post-COVID Model: Relevance of Chemokine-Mediated Inflammation

**DOI:** 10.3390/v17121629

**Published:** 2025-12-16

**Authors:** Silvia Flaj-Prados, Esperanza Herradón Pliego, Carlos Goicoechea Garcia, Eva M. Sánchez-Robles, Lars Arendt-Nielsen, César Fernández-de-las-Peñas, Visitación López-Miranda

**Affiliations:** 1 Department of Basic Health Sciences, Universidad Rey Juan Carlos, 28922 Madrid, Spain; silvia.flaj@urjc.es (S.F.-P.); esperanza.herradon@urjc.es (E.H.P.); carlos.goicoechea@urjc.es (C.G.G.); eva.sanchez@urjc.es (E.M.S.-R.); visitacion.lopezmiranda@urjc.es (V.L.-M.); 2 Unidad Asociada al Instituto de Química Medica (IQM) del Consejo Superior de Investigaciones Científicas (CSIC), Universidad Rey Juan Carlos, 28922 Madrid, Spain; 3 High Performance Research Group in Experimental Pharmacology (Pharmakom-URJC), Universidad Rey Juan Carlos, 28922 Madrid, Spain; 4 Center for Neuroplasticity and Pain (CNAP), Sensory-Motor Interaction (SMI) Center, Department of Health Science and Technology, Faculty of Medicine, Aalborg University, DK-9220 Aalborg, Denmark; lan@hst.aau.dk; 5 Department of Gastroenterology & Hepatology, Mech-Sense, Clinical Institute, Aalborg University Hospital, DK-9000 Aalborg, Denmark; 6 Steno Diabetes Center North Denmark, Clinical Institute, Aalborg University Hospital, DK-9000 Aalborg, Denmark; 7 Department of Physical Therapy, Occupational Therapy, Physical Medicine and Rehabilitation, Universidad Rey Juan Carlos, 28922 Madrid, Spain

**Keywords:** post-COVID, hACE2 mouse, cardiac inflammation, post-acute sequelae of SARS-CoV-2 infection

## Abstract

Post-COVID cardiac complications have emerged as a significant and persistent clinical concern, yet their underlying mechanisms remain poorly understood. Animal models can act as proxies to investigate the pathophysiology of the human, post-acute sequelae of SARS-CoV-2 infection (PASC). The aim of this experimental study was to evaluate the expression of inflammatory biomarkers in cardiac tissue 28 days after SARS-CoV-2 infection in a female hACE2 mouse model, with a focus on chemokine-mediated immune activation. Twelve female C57BL/6 hACE2 mice were infected with the Omicron variant (BA.1.17 lineage) of SARS-CoV-2, and eleven non-infected mice served as controls. Cardiac tissue was analyzed via Western blot for markers of innate immune activation (TLR4, MyD88, NF-κB) and pro-inflammatory cytokines (IL-6, IL-18, IL-1β, TNF-α, CD11d). Cardiac tissue injury markers (iNOS, PAI-1 and Connexin43) were also analyzed. Compared to non-infected mice, cardiac tissue from infected mice showed significantly higher expression of IL-6 (*p* = 0.028), indicating an inflammatory state, and CD11d (*p* = 0.016), suggesting an inflammatory stage accompanied by sustained activation of chemokine-mediated inflammatory signaling. No significant differences in TLR4 (*p* = 0.340), MyD88 (*p* = 0.410), NF-κB p65 (*p* = 0.780), IL-18 (*p* = 0.548), IL-1β (*p* = 0.455), and TNF-α (*p* = 0.125) expressions were observed Similarly, no changes in cardiac damage markers (iNOS: *p* = 0.4684; PAI-1: *p* = 0.5345; Connexin 43: *p* = 0.2879) were found. The results of this experimental study would support the hypothesis of persistent low-grade inflammation as a contributor to post-COVID cardiac sequelae in females that is not accompanied by severe tissue damage, as also observed in clinical studies. This study also reinforces the need for studies evaluating the functional and structural evolution of the myocardium after an acute SARS-CoV-2 infection.

## 1. Introduction

The outbreak caused by the coronavirus disease 2019 (COVID-19) pandemic has led to one of the most relevant health crises of the current century. The Severe Acute Respiratory Syndrome Coronavirus 2 (SARS-CoV-2), the pathogen causing COVID-19, mainly affects the respiratory system; however, it has become evident that the disease can affect multiple systems and organs [1].

Clinically, multi-organ affectations explain the plethora of long-lasting persistent symptoms (e.g., cardiovascular, neurological, respiratory, musculoskeletal) experienced after the acute COVID-19 phase [2]. Evidence suggests that up to 25–30% of COVID-19 survivors report long-lasting symptoms two or three years after the infection [3,4]. The presence of long-lasting symptoms after an acute SARS-CoV-2 infection has received several names [5], but post-COVID-19 condition is the term proposed by the World Health Organization (WHO) [6]. By definition, post-COVID-19 condition cannot be explained by any medical condition [6]; however, it has been observed that several COVID-19 survivors exhibit long-lasting damage in the cardiovascular or gastrointestinal systems, a phenomenon specifically defined as Post-Acute Sequelae of COVID-19 (PASC) [7]. Among the long-lasting PASC, cardiac complications have risen as relevant healthcare problems due to their high associated burden [8]. Different meta-analyses have found that almost 25% of COVID-19 survivors report at least one cardiovascular symptom two or three years after infection: fatigue (28%, 95%CI 12% to 47%), chest pain (22%, 95%CI 14% to 33%), palpitations (18% 95% CI 13% to 24%) or dyspnea (12%, 95%CI 10% to 15%) [4,9].

The mechanisms behind post-COVID cardiac symptoms are complex, and several hypotheses, including endothelial dysfunction, myocardial inflammation, and autonomic dysregulation, have been proposed [10]. In this context, underlying inflammation has emerged as a key mechanism in the development of post-COVID cardiac sequelae. Recent evidence suggests that cardiac inflammation may persist long after acute SARS-CoV-2 infection, even in the absence of active viral replication [11]. Thus, trace-level cytokines have been shown to impair cardiomyocyte function in patients with post-acute sequelae of COVID-19. This highlights a potential mechanism for cardiovascular symptoms in PASC independent of direct viral presence [11]. This sustained inflammation may contribute to endothelial dysfunction, vascular remodeling, and tissue damage, potentially explaining symptoms such as palpitations, dyspnea or chest pain. Furthermore, the dissociation between clinical symptoms and objective findings in cardiac tests suggests that non-classical immune mechanisms, such as localized chemokine signaling, may be involved in the pathophysiology of these manifestations [12]. Preclinical studies have also revealed an important role of β2 integrins in the pathophysiology of cardiac injury, exacerbating cardiac inflammation when their expression is altered [13]. Among them, CD11d/CD18 facilitates macrophage retention within inflamed tissues, contributing to the persistence of chronic inflammation and the development of vascular lesions such as atherosclerosis [14,15]. Although the specific role of CD11d in SARS-CoV-2 infection has not been previously characterized, available evidence suggests that it may contribute to cardiac damage driven by exacerbated inflammatory processes in COVID-19.

Understanding of the underlying mechanisms behind long-lasting cardiac complications remains limited, partly due to the difficulty of studying organ damage in living humans and to the observed dissociation between clinical symptomatology and objective measures of cardiac function. In this context, animal models have become fundamental tools to investigate the pathogenesis of PASC, allowing controlled experimental studies of the disease and facilitating the identification of structural or functional changes in specific organs [16]. Different animal models, including non-human primates, golden Syrian hamsters, transgenic or virus-adapted mice, have been used for studying viral persistence, immune dysfunction, intestinal dysbiosis, and neurovascular changes that characterize PASC [17]. Interestingly, there are few studies investigating the impact of SARS-CoV-2 on cardiac function or structure [18]. In fact, most studies using animal models have evaluated the cardiovascular alterations at the acute phase of the infection, reporting that SARS-CoV-2 induces cardiac damage, including myocarditis, increased microthrombi or areas of damaged or necrotic heart tissue [19,20]. To date, no study has assessed the post-acute impact of SARS-CoV-2 infection on cardiac function or integrity in an experimental animal model [18].

Jiménez de Oya et al. described an animal model by using hACE2 female multigene mice C57BL/6J infected with Omicron (lineage BA.1.17) SARS-CoV-2 variant [21]. This mouse model showed detectable viral SARS-CoV-2 RNA and significantly elevated levels of pro-inflammatory interleukins (e.g., IL-6) and tumor necrosis factor-alpha (TNF-a) in the lung tissues 4 days, but not 15 days, after infection [21]. Therefore, studies examining cardiac alterations following acute SARS-CoV-2 infection using this murine model can provide valuable insights that may contribute to a better understanding and management of long-term post-COVID cardiac sequelae in patients.

Accordingly, the present study aimed to investigate the presence of a pro-inflammatory profile in cardiac tissue using the abovementioned post-COVID murine model. This approach will enable an exploration of the pathophysiological mechanisms underlying cardiac complications associated with conditions such as PASC. Given that most experimental studies on COVID-related cardiac symptoms have been conducted in male animals, this study utilizes a female animal model to confirm these alterations, particularly in underrepresented populations such as females. The study may provide hints to further explore the causes of post-COVID cardiac symptoms in patients.

## 2. Methods

### 2.1. Ethics Statement

This study was conducted in accordance with the Ethical Committee of Animal Experimentation of INIA-CSIC and by the Division of Animal Protection of the Comunidad de Madrid (PROEX 115.5-21). It followed the guidelines for the Care and Use of Laboratory Animals of the European Community (European Directive 2010/63/EU). Thus, the local Research Ethics Committee of Universidad Rey Juan Carlos (Madrid, Spain, code: ENM159/202311202122021, date of approval: 22 January 2022) approved the study design.

### 2.2. Model of SARS-CoV-2 Infection in Female Mice

A sample of C57BL/6 female mice was used for transgene production, i.e., transgenic mice expressing the human angiotensin-converting enzyme 2 (ACE2) receptor under the human cytokeratin 18 (K18) gene promoter’s control (hACE2) [22]. Subsequently, 6-week-old, 20–25 g, female hACE2 mice were anesthetized with 5–2% of isoflurane (+0.5–1 L/min) and intranasally inoculated with 50 μL of the Omicron variant of SARS-CoV-2 (lineage BA.1.17, 104 TCID50/mouse) as previously described [21]. All experimental procedures related to the animal model were conducted in the biosafety level 3 facilities at the Instituto Nacional de Investigación y Tecnología Agraria y Alimentaria (INIA-CSIC), Madrid (Spain).

### 2.3. Experimental Study Design

**Figure 1:**
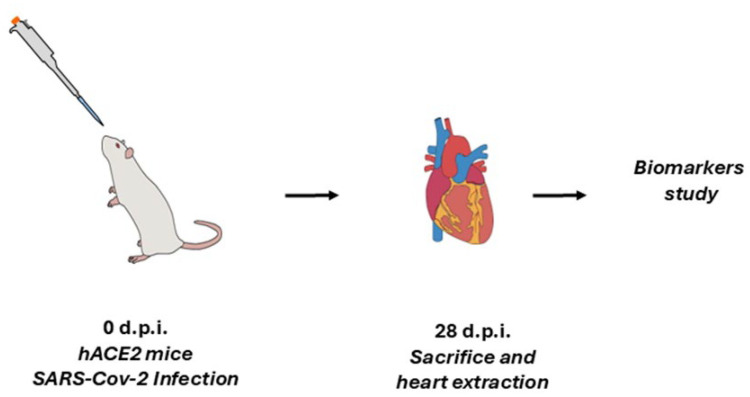
Experimental protocol.

An initial sample of twelve (*n* = 12) hACE2 female mice infected with the SARS-CoV-2 Omicron variant (infected group) and eleven (*n* = 11) uninfected hACE2 animals were used. All mice received food and water *ad libitum* and were monitored daily for clinical signs and body weight. Animals that showed advanced clinical features of SARS-CoV-2 infection and a significant deterioration in their health status (e.g., weight loss exceeding 20% of initial body weight or severe symptoms such as lethargy, inactivity, hunched posture, or markedly abnormal respiration) were anesthetized and sacrificed by cervical dislocation. All animals that survived and reached the end of the experiment (28 days post-infection) were sacrificed, and cardiac tissue was obtained for biological study (Figure 1). The period of 28 days post-infection was chosen because it correlates to 3 years in the human lifespan, which corresponds to a post-COVID condition [23].

### 2.4. Western Blot Analysis

Following mouse sacrifice, cardiac tissue was rapidly dissected and immediately frozen at −80 °C. Cardiac tissue was homogenized in ice-cold RIPA buffer supplemented with a protease inhibitor cocktail (Roche, Spain) and using a TissueLyser II system (Qiagen, Hilden, Germany) for protein extraction. All homogenates were centrifuged, and the supernatant was extracted. Total protein concentrations were determined using the NanoDrop 2000c spectrophotometer (Thermo Fisher Scientific, Inc., Waltham, MA, USA). Protein expression analysis by Western blot was performed using cardiac tissue from 7 surviving animals in the infected group and a similar sample in the uninfected group

For electrophoresis, 40 µg of protein from the heart was loaded onto a precast Mini-Protean^®^ TGXTM gel (Bio-Rad, Madrid, Spain) and transferred to a polyvinylidene fluoride (PVDF) membrane (Bio-Rad, Madrid, Spain). Membranes were blocked with 3% non-fat dry milk at room temperature for 1h and then incubated at 4° overnight with the primary following antibodies: Toll-like receptor-4 (TLR4) 1:500 (Novus Biosciences, Toronto, ON, Canada, Cat#NB100-56566); Myeloid differentiation primary response gene 88 (MyD88) 1:500 (Abcam, Cambridge, UK, Cat#2064); Nuclear Factor kappa-light-chain-enhancer of activated B cells (NF-κβ p65) 1:1000 (Abcam, Cat#16502); tumour necrosis factor-alpha (TNF-α) 1:1000 (Abcam, Cambridge, UK, Cat#34674); IL-6 1:500 (Abcam, Cat#290735); IL-1β 1:1000 (Abcam, Cambridge, UK, Cat#283818); IL-18 1:750 (Invitrogen, Waltham, MA, USA, Cat#PA5-79482); integrin alpha D (CD11d) 1:1000 (Abcam, Cambridge, UK, Cat#231534); iNOS 1:2000 (Invitrogen, Waltham, MA, USA, Cat#PA3-030A); PAI-1 1:500 (Abcam, Waltham, MA, USA, Cat#7205); Connexin 43 1:10,000 (Cell Signaling Technology, Waltham, MA USA, Cat#3512). After primary incubation, membranes were incubated for 1 h at room temperature with the specific secondary antibody (Goat anti-Mouse IgG (H+L) Secondary Antibody, HRP (Thermo Fisher Scientific, Inc., Waltham, MA USA, Cat#31430) or goat anti-rabbit IgG (H+L) secondary antibody, AP (Thermo Fisher Scientific, Inc., Waltham, MA USA, Cat#31340). Glyceraldehyde-3-phosphate dehydrogenase (GAPDH) 1:5000 (Abcam, Waltham, MA, USA, Cat#8245) was used as a loading control with secondary antibody (1:10,000).

Protein bands were visualized using the Clarity™ Western ECL substrate (Bio-Rad^®^, Madrid, Spain) and detected with a Chemidoc™ XRS+ system or ChemiDoc™ MP Imaging System. Band intensities were analyzed via densitometry using ImageLab™ software 3.0 (Bio-Rad^®^, Madrid, Spain) and normalized to GAPDH.

### 2.5. Statistical Analysis

Statistical analyses were conducted using GraphPad Prism software, version 8.0 (San Diego, CA, USA). The normality of quantitative data was assessed using the D’Agostino-Pearson test. Results are presented as mean ± standard error of the mean (S.E.M.). Biomarker levels were compared using either an unpaired Student’s *t*-test or the Mann–Whitney U test, depending on the distribution of the data. In cases where outliers were detected, they were omitted for statistical comparison.

## 3. Results

Five female mice infected with the SARS-CoV-2 Omicron variant died (*n* = 5/12, 58.5% survival rate), whereas no female mice in the non-infected group died (*p* = 0.017, Log-rank Mantel–Cox test).

### 3.1. Cardiac Expression of the TLR4/MyD88/NF-κB Signaling Pathway

**Figure 2:**
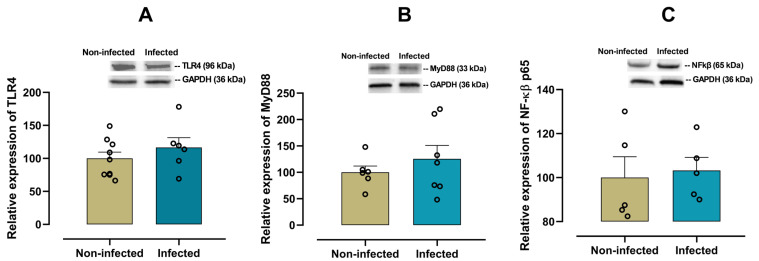
Protein expression of the TLR4 pathway in cardiac tissue from infected and non-infected female mice. Representative immunoblot and bar graph of the corresponding densitometric analysis of TLR4 (**A**) (*n* = 9 for non-infected; *n* = 6 for infected), MyD88 (**B**) (*n* = 6 for non-infected; *n* = 7 for infected) and NF-κB p65 (**C**) (*n* = 5 for non-infected; *n* = 5 for infected) in cardiac tissue from experimental female groups. Protein levels were normalized to GAPDH. Data are expressed as mean ± S.E.M. *n* represents biologically independent assays. All data from the graphs followed a normal distribution, and statistical comparisons were performed using an unpaired Student’s *t*-test.

To explore the involvement of innate immune activation in cardiac inflammation, we analyzed the expression of the TLR4/MyD88/NF-κB signaling pathway in the heart. No significant differences in TLR4 (*p* = 0.340, Figure 2A), MyD88 (*p* = 0.410, Figure 2B) or NF-κB p65 (*p* = 0.780, Figure 2C) expression levels in cardiac tissue between infected and non-infected mice were observed.

### 3.2. Cardiac Expression of Pro-Inflammatory Cytokines and Chemokines

**Figure 3:**
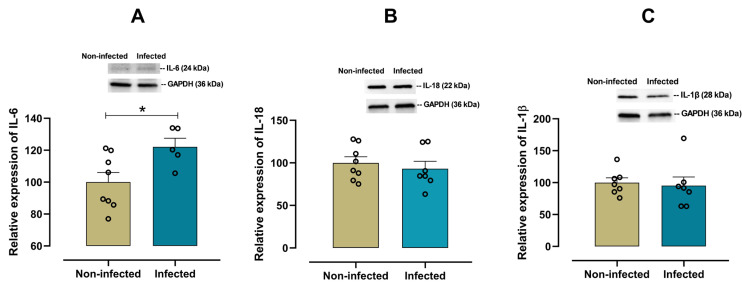
Protein expression of IL-6, IL-18, and IL-1β in cardiac tissue from infected and non-infected female mice. Representative immunoblot and bar graph of the corresponding densitometric analysis of IL-6 (**A**) (*n* = 8 for non-infected; *n* = 5 for infected), IL-18 (**B**) (*n* = 8 for non-infected; *n* = 7 for infected), and IL-1β (**C**) (*n* = 7 for non-infected; *n* = 7 for infected) in cardiac tissue from experimental female groups. Protein levels were normalized to GAPDH. Data are expressed as mean ± S.E.M. *n* represents biologically independent assays. A and B graph data followed a normal distribution, and an unpaired Student’s *t*-test was used for statistical comparisons. C graph data followed a non-normal distribution, and a non-parametric Mann–Whitney test was performed, * *p* < 0.05 vs. non-infected group.

**Figure 4:**
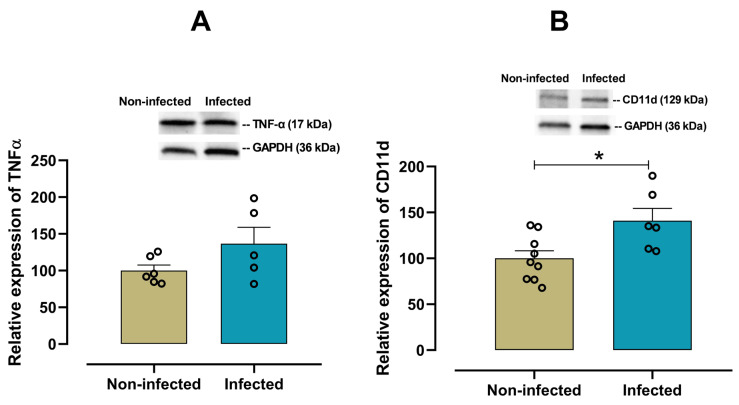
Protein expression of TNF-α and CD11d in cardiac tissue from infected and non-infected female mice. Representative immunoblot and bar graph of the corresponding densitometric analysis of TNF-α (**A**) (*n* = 6 for non-infected; *n* = 5 for infected), and CD11d (**B**) (*n* = 9 for non-infected; *n* = 6 for infected) in cardiac tissue from experimental female groups. Protein levels were normalized to GAPDH. Data are expressed as mean ±S.E.M. n represents biologically independent assays. All data from the graphs followed a normal distribution, and statistical comparisons were performed using an unpaired Student’s *t*-test, * *p* < 0.05 vs. non-infected group.

To characterize the cardiac pro-inflammatory profile in infected female mice, the expression of IL-6, IL-18, IL-1β, TNF-α and CD11d was examined. A significant increase (*p* = 0.028) in the pro-inflammatory cytokine IL-6 was observed in infected female mice when compared with non-infected mice (Figure 3A). In contrast, there were no significant differences in the expression of cardiac IL-18 (*p* = 0.548, Figure 3B), IL-1β (*p* = 0.455, Figure 3C), and TNF-α (*p* = 0.125, Figure 4A) between infected and non-infected mice. Additionally, a significant increase (*p* = 0.016) in CD11d expression (Figure 4B) was also observed in infected female mice, suggesting a sustained activation of the chemokine-mediated inflammatory signaling in cardiac tissue, potentially reflecting leukocyte retention and prolonged immune cell presence.

### 3.3. Correlation Between the Expression of Cardiac IL-6 and CD11d

**Figure 5:**
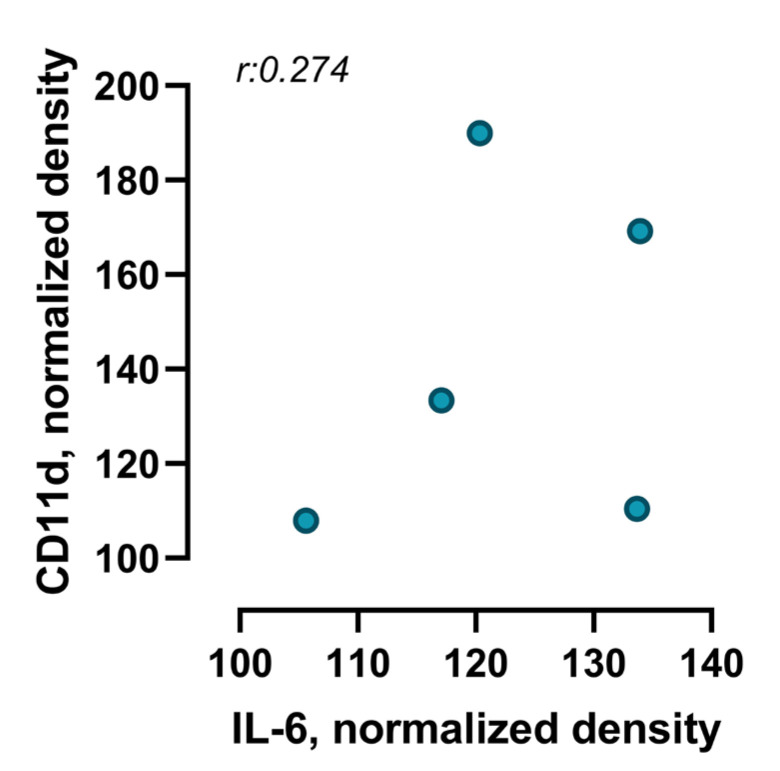
Correlation between IL-6 and CD11d expression levels in cardiac tissue of infected animals over a period of 28 days after infection. Scatter plot illustrates the correlation between IL-6 and CD11d levels in cardiac tissue of mice in a post-COVID condition. Each point represents an individual animal (*n* = 5 IL-6; *n* = 6 CD11d). Pearson’s correlation coefficient (r: 0.274; *p* = 0.655) indicates a very weak and non-significant positive correlation between both proteins.

Although both IL-6 and CD11d were individually found to be significantly upregulated in the cardiac tissue of infected mice at 28 days post-infection compared to non-infected controls, Pearson correlation analysis showed no significant linear relationship between their expression levels. The correlation coefficient (r = 0.274), coefficient of determination (R2 = 0.075) and *p*-value (*p* = 0.655) indicate a small and non-significant association (Figure 5).

### 3.4. Cardiac Expression of iNOS, PAI-1 and Connexin-43 as Markers of Cardiac Tissue Damage

To analyse the possible existence of damage to cardiac tissue, measurements of iNOS, PAI-1 and connexin 43 (Cx43) were included. iNOS reflects inflammatory activation and oxidative stress, PAI-1 is associated with fibrinolytic alterations and risk of fibrosis, while Cx43 is essential for electrical communication between cardiomyocytes. Changes in the expression of these markers have been linked to tissue damage in chronic inflammatory processes [24,25,26].

**Figure 6:**
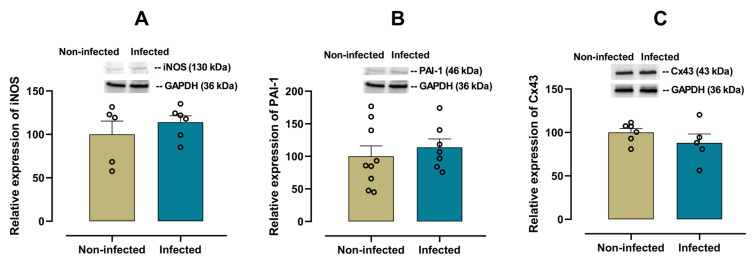
Protein expression of iNOS, PAI-1 and Connexin 43 (Cx43) in cardiac tissue from infected and non-infected female mice. Representative immunoblot and bar graph of the corresponding densitometric analysis of iNOS (**A**) (*n* = 5 for non-infected; *n* = 6 for infected), PAI-1 (**B**) (*n* = 9 for non-infected; *n* = 7 for infected) and Cx43 (**C**) (*n* = 6 for non-infected; *n* = 5 for infected) in cardiac tissue from experimental female groups. Protein levels were normalized to GAPDH. Data are expressed as mean ± S.E.M. *n* represents biologically independent assays. All data from the graphs followed a normal distribution, and statistical comparisons were performed using an unpaired Student’s *t*-test.

No significant differences in the expression of iNOS (*p* = 0.4084, Figure 6A) and PAI-1 (*p* = 0.5345, Figure 6B) or cardiacCx43 (*p* = 0.2879, Figure 6C) were observed between infected and non-infected mice.

## 4. Discussion

The current experimental study using C57BL/6 female hACE2 mice infected with SARS-CoV-2 Omicron variant provides evidence on IL-6 and CD11d overexpression in cardiac tissue, 28 days after the infection (post-COVID condition). In contrast, TNF-α, IL-1β, and IL-18 levels remained within normal ranges, showing no significant differences between infected and non-infected animals. This selective inflammatory profile suggests a shift from acute systemic inflammation to a localized and sustained immune activation in the heart at a post-COVID stage. In addition, to our knowledge, this is the first study describing the involvement of β2 integrin and CD11d in cardiac damage in post-COVID-19 condition. On the other hand, it should be noted that, although no serious tissue damage was found in the cardiac tissue in this study, the inflammatory microenvironment could cause it in the long term, reinforcing the need for studies evaluating the functional and structural evolution of the myocardium. These results may contribute to understanding post-COVID cardiac damage and support the need for personalized approaches in the management of post-acute sequelae in patients with long-lasting cardiac symptoms.

Current results align with the hypothesis that post-COVID cardiac sequelae may be mediated by immune dysregulation rather than direct viral cytotoxicity, as supported by autopsy studies showing immune cell infiltration without viral presence [27]. These findings are also consistent with recent studies showing that persistent cardiac inflammation may be driven by non-classical immune pathways, particularly in the absence of detectable viral RNA in cardiac tissue [28]. Importantly, the use of a female animal model in the current study addresses a critical gap in the literature, as most preclinical COVID-19 studies have focused on male animals, despite evidence of sex-specific differences in immune response and cardiovascular outcomes [29].

### 4.1. TLR4/MyD88/NF-κB Pathway in Post-Acute Sequelae of COVID-19

The TLR4/MyD88/NF-κB axis has been widely implicated in myocardial inflammation across various conditions, including viral infections. However, in our post-COVID model, no significant differences were observed in the expression of TLR4, MyD88, or NF-κB p65 in cardiac tissue, 28 days after SARS-CoV-2 infection, suggesting that this pathway may not be involved in the sustained cardiac inflammation observed post-infection. Moreover, the absence of TLR4 pathway activation would suggest that the inflammatory response is not driven by pathogen-associated molecular pattern (PAMP) recognition or acute cytokine storm, but rather by localized chemokine-mediated signaling. This aligns with findings from human studies showing persistent myocardial injury and microthrombi in COVID-19 survivors, even in the absence of active viral replication [30,31].

Recent studies have shown that, although the SARS-CoV-2 spike protein can activate TLR4 in human cardiac cells [32] during the acute phase or in the presence of active viral components, this does not correspond to the post-infectious state of our model. Structural investigations have confirmed that the S1 domain of the viral spike interacts with the TLR4/MD2 complex, promoting inflammatory signaling [33], but this interaction seems to depend on the active presence of the virus or circulating viral fragments, which may explain the lack of activation observed in our animal model at 28 days post-infection. It is important to note that other studies in animal models have shown that activation of the TLR4/MyD88/NF-κB axis can induce significant cardiac inflammation in conditions such as acute myocardial infarction or post-traumatic stress [34,35], but these models differ from the post-infectious context without active viral replication that characterizes the SARS-CoV-2 infection model. These findings reinforce the idea that post-COVID cardiac inflammation may be mediated by mechanisms distinct from classical PAMP recognition, such as those activating TLR4. Instead, it may involve inflammation induced by persistent tissue damage, endothelial dysfunction, or endogenous danger signals (DAMPs), opening new avenues for the study of therapies targeting these mechanisms.

### 4.2. Cardiac Inflammation in Post-Acute Sequelae of COVID-19

Regarding cytokines, the overexpression of IL-6 and CD11d in our study reinforces the hypothesis of localized and sustained inflammation in a post-COVID phase. In relation to IL-6, the COVID-19 pandemic revealed various long-term cardiovascular complications linked to increased inflammatory responses, particularly through the IL-6 activity [36]. High levels of IL-6 during and after an acute SARS-CoV-2 infection are linked with poor outcomes, such as acute respiratory distress syndrome, myocarditis, endothelial dysfunction, and thrombotic events [31,37,38,39]. It is important to highlight that human studies have assessed IL levels in the plasma but not in specific organs. Our study would confirm an overexpression of IL-6 specifically within the cardiac tissue, supporting the hypothesis that SARS-CoV-2 directly affects the heart, potentially leading to endothelial injury, inflammatory cytokine storm syndrome, hypercoagulability, or thrombosis. Therefore, the observed overexpression of IL-6 and CD11d levels (see below) in the cardiac tissue provides a potential substrate that explains the hypothesis of endothelial dysfunction proposed for explaining post-COVID cardiac problems [40].

In the present research, at 28 days post-infection, CD11d expression was markedly elevated in cardiac tissue, whereas levels of TNF-α, IL-1β, and IL-18 remained within the normal range. This dissociation suggests that CD11d upregulation may occur independently of classical innate immune activation or may reflect a persistent, non-canonical inflammatory mechanism. CD11d, an integrin involved in leukocyte adhesion and retention, is typically upregulated in chronic inflammatory environments and may reflect sustained immune cell presence in cardiac tissue identified [41]. Although direct evidence of CD11d involvement in post-COVID-19 cardiac tissue is currently lacking, previous studies have demonstrated the role of related integrins, such as CD11b, in macrophage retention and vascular remodeling in COVID-affected hearts. Werlein et al. conducted a comprehensive multimodal analysis of cardiac autopsy samples from COVID-19 patients and identified a distinct angiocentric, macrophage-driven inflammatory process [42]. Their findings revealed a significant infiltration of CD11b^+^/TIE2^+^ macrophages in perivascular regions, which were associated with intussusceptive angiogenesis—a non-sprouting form of vascular remodeling-suggesting a mechanism of sustained immune cell presence and tissue-specific inflammation [42]. These macrophages were notably elevated in patients hospitalized more than 10 days after infection onset, and their presence correlated with increased expression of angiogenic and hypoxia-related genes such as VEGFA, FGF2, and HIF1. Given the functional similarities within the β2 integrin family, the observed CD11d overexpression in our model may reflect a comparable mechanism of sustained immune cell presence and tissue-specific inflammation. This highlights the need to further investigate sex-specific and non-specific pathways involved in post-COVID cardiovascular sequelae, which may inform more targeted therapeutic strategies.

Despite the well-established role of IL-1β, IL-18, and TNF-α in acute inflammatory responses, our study did not observe significant changes in their expression in cardiac tissue 28 days post SARS-CoV-2 infection. This finding suggests that these cytokines may not be central mediators of sustained cardiac inflammation in the post-acute COVID-19 phase. IL-1β is a key pro-inflammatory cytokine involved in early immune response and has been implicated in acute myocarditis and systemic inflammation during COVID-19 [43]. However, its absence in our model aligns with recent findings showing that IL-1β levels tend to normalize in convalescent stages, even in patients with persistent symptoms [44]. This may reflect a resolution of the acute inflammatory phase or a shift toward alternative immune pathways. Interleukin-18 (IL-18), another inflammasome-related cytokine, has also been associated with myocardial inflammation in both COVID-19 infection and vaccine-related myocarditis [45]. Interestingly, while IL-18 has been shown to correlate with cardiac edema in convalescent patients [46], our model did not replicate this elevation, possibly due to differences in timing, viral variants, or tissue-specific immune regulation. It is also possible that IL-18 expression is more prominent in systemic circulation or pulmonary tissue than in the heart tissue during the post-acute phase. TNF-α, a central mediator of cytokine storm and acute cardiac injury, has been widely studied in COVID-19 pathophysiology. Elevated TNF-α levels have been linked to acute myocardial damage and activation of the NF-κB pathway [47]. However, in our model, TNF-α expression remained unchanged, suggesting that the TNF-NF-κB axis may not be persistently activated in cardiac tissue after viral clearance. This supports the hypothesis that post-COVID cardiac sequelae may be driven by non-classical inflammatory mechanisms, such as chemokine-mediated leukocyte retention or endothelial dysfunction, rather than sustained TNF-α signaling. Overall, the lack of significant changes in IL-1β, IL-18, and TNF-α expression in cardiac tissue highlights the complexity of post-COVID immune responses and underscores the importance of investigating alternative pathways beyond classical cytokine storm mediators.

Our findings show that, despite the upregulation of both IL-6 and CD11d in the cardiac tissue of infected mice, there was no significant linear correlation between the markers. This result is consistent with clinical evidence in post-COVID patients, where elevated IL-6 has been associated with myocardial damage and myocarditis, but without a clear relationship with other specific immune markers such as CD11d [46,48,49]. Furthermore, the lack of correlation observed in our model suggests that IL-6 and CD11d may be regulated by independent pathways, which is consistent with recent reviews highlighting the complexity of post-COVID immune mechanisms, where multiple cells and mediators participate in a non-linear manner in cardiac injury [37,50]. These findings reinforce the need for further studies exploring the interaction between cytokines and adhesion molecules in post-COVID cardiovascular pathophysiology, as well as their value as potential prognostic biomarkers.

### 4.3. Cardiac Tissue Damage in Post-Acute Sequelae of COVID-19

Myocardial overexpression of PAI-1 is recognised as a profibrotic marker that promotes ventricular remodelling and fibrosis, contributing to cardiac dysfunction [51,52,53]. Similarly, increased iNOS is associated with oxidative stress and contractile impairment, exacerbating myocardial damage [54] and alterations in the expression or distribution of connexin 43 compromise electrical conduction, increase susceptibility to arrhythmias, and accelerate progression to heart failure [55]. In our study, the expression of PAI-1 and iNOS in cardiac tissue from female mice infected with SARS-CoV-2 showed modest increases (≈13%) compared to noninfected mice. This finding suggests that, in this PASC condition, there is no marked activation of inflammatory (iNOS) or fibrinolytic (PAI-1) pathways, which could indicate an absence of severe myocardial damage. These results are consistent with the hypothesis that the cardiac inflammatory response induced by SARS-CoV-2 is more pronounced in the acute phase and tends to normalize in later stages [56,57], reinforcing the need for studies evaluating the functional and structural evolution of the myocardium.

### 4.4. Limitations

Thus, some limitations of this study could be pointed out. The relatively low number of animals may also reduce statistical power and generalizability of the findings. A second limitation of this study is that cytokine analysis was performed using Western blot rather than ELISA, which offers higher sensitivity and absolute quantification. However, the sample preparation protocol (homogenization in lysis buffer) precluded ELISA analysis. Future research should incorporate ELISA or multiplex immunoassays to complement protein expression data. Another limitation could be the absence of histological and immunofluorescence analyses of cardiac tissue, which would have provided valuable information on structural and cellular alterations following PASC.

Finally, it should be noted that sex has a greater influence on the initiation and progression of the COVID-19 disease. In fact, the differences in severity and mortality could be explained by the hormonal and genetic regulation [58]. Our study was conducted in females of the K18-hACE2 transgenic mouse model. This model is widely used to study SARS-CoV-2 infection, as it expresses the human ACE2 receptor under the control of the cytokeratin-18 promoter, allowing for efficient viral entry and replication [59]. However, this overexpression could not replicate the physiological regulation of ACE2, including modulation by sex hormones such as estradiol, which may partially mask their influence on susceptibility and disease progression. Future studies should incorporate sex comparisons and hormonal modulation to better understand these differences.

## 5. Conclusions

This preclinical mouse study demonstrates that SARS-CoV-2 infected animals show a selective inflammatory response in cardiac tissue, 28 days post-infection. The significant overexpression of IL-6 and CD11d, and the absence of elevated TNF-α, IL-1β, IL-18, and TLR4/MyD88/NF-κB pathway activation, suggest a non-classical, localized immune activation. These findings support the hypothesis of sustained chemokine-mediated inflammation in the heart, contributing to long-term cardiac sequelae in post-COVID conditions.

Further, although no serious tissue damage has been found in the cardiac tissue in SARS-CoV-2-infected animals, the inflammatory microenvironment could cause damage in the long term, reinforcing the need for long-lasting studies evaluating the functional and structural evolution of the myocardium.

## Data Availability

All data is presented in the text.
